# A Recyclable and Biodegradable High‐Performance Piezoelectric Nanogenerator for Self‐Powered Biosensing and Assistive Human‐Machine Interaction

**DOI:** 10.1002/advs.77780

**Published:** 2026-09-27

**Authors:** Tingshuai Luo, Tian Xiao, Luyu Zhang, Bailang Zhang, Shengchang Lu, He Xiao, Jianguo Li, Kai Liu, Chaoji Chen, Hui Wu

**Affiliations:** ^1^ College of Material Engineering Fujian Agriculture and Forestry University Fuzhou People's Republic of China; ^2^ Hubei Biomass‐Resource Chemistry and Environmental Biotechnology Key Laboratory Hubei Provincial Engineering Research Center of Emerging Functional Coating Materials School of Resource and Environmental Sciences Wuhan University Wuhan People's Republic of China

**Keywords:** biodegradable, cellulose nanofibers, flexible electronics, PENG, sensors

## Abstract

The escalating global challenge of electronic waste demands energy‐harvesting materials that deliver high performance without compromising environmental sustainability. We present a molecularly engineered, fully biodegradable piezoelectric nanogenerator (PENG) based on a composite of 2,2,6,6‐tetramethylpiperidine‐1‐oxyl (TEMPO)‐oxidized cellulose nanofibers (TOCNF) and 2,2,3,3,4,4‐hexafluoropentane‐1,5‐diol (HFPD). Multiscale hydrogen bonding orchestrates a hierarchically ordered structure, yielding an exceptional piezoelectric coefficient of 14.0 pC·N^−1^ and excellent fatigue durability (100 000 bending cycles). This PENG exhibits high sensitivity for real‐time monitoring of human physiological signals, from limb movements and joint kinematics to speech and respiration. Integrated with a deep learning framework, the system achieves 98.7% accuracy in recognizing Morse code signals, offering a novel assistive communication tool. In vivo experiments demonstrate its capability to capture limb motion and respiratory signals, confirming its potential for implantable biomonitoring. Crucially, the device is fully recyclable in water without performance loss and biodegrades in soil within 25 days. This work provides a scalable design strategy for functional materials that unify high performance with full lifecycle sustainability, charting a transformative path toward environmentally benign electronics.

## Graphical abstract

By exploiting multiscale hydrogen‐bonding interactions between HFPD and TOCNF, the composite simultaneously achieves a high piezoelectric coefficient (14.0 pC·N^−1^), outstanding flexibility (100 000 bending cycles), water‐assisted recyclability, excellent biodegradability, and sensitive wearable/implantable sensing for motion, speech, and Morse code recognition.

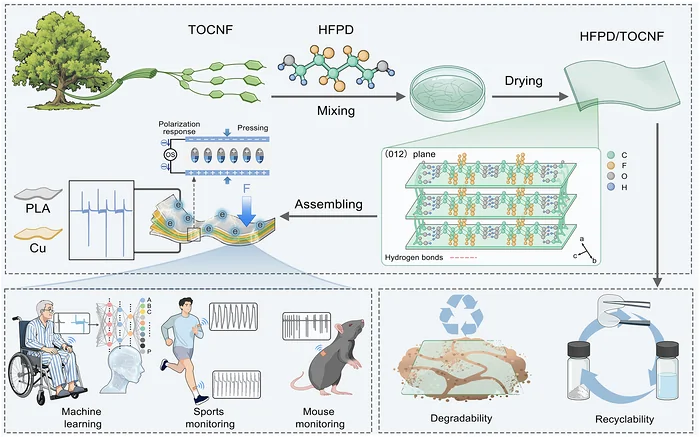

## 1 Introduction

Electronic devices are integral to modern technological systems, driving essential functions across a broad range of sectors and facilitating technological advancements [1]. However, the extensive use and rapid turnover of these devices have given rise to a global e‐waste crisis [2]. Accelerated product lifecycles, limited functional longevity, and wear and tear are key contributors to rising disposal rates, with approximately 50 million tons of e‐waste generated each year globally [3]. According to the United Nations Environment Programme, e‐waste accumulation is increasing at a rate of about 5% annually [4]. The 2024 Global E‐Waste Monitor report further indicates that the volume of recyclable e‐waste remains far below the volume generated. This waste contains hazardous substances, such as heavy metals and persistent organic pollutants, which cause significant environmental damage to soil and aquatic ecosystems and pose risks to human health through bioaccumulation [5]. Beyond environmental concerns, ineffective e‐waste management also hinders the progress of circular economies and social sustainability. The recoverable value within global e‐waste was estimated at 57 billion USD in 2020, with projections suggesting it will rise to 65.8 billion USD by 2026 [6, 7]. Consequently, the development of sustainable e‐waste management strategies has become an urgent global priority.

To address the environmental challenges posed by electronic waste, research is increasingly focused on developing biodegradable and recyclable materials to replace conventional electronic components [8]. In the field of mechanical sensing, various energy conversion mechanisms have been explored using eco‐friendly materials, such as piezoresistive [9, 10, 11], piezocapacitive [12, 13, 14], triboelectric [15, 16], and piezoelectric [17, 18] effects. While piezoresistive and piezocapacitive devices offer high sensitivity, they rely on external power sources, complicating system design and reducing energy efficiency. In contrast, triboelectric and piezoelectric effects convert mechanical energy into electricity, enabling self‐powered systems [19]. However, triboelectric generators typically require complex microstructural designs for optimal performance. Piezoelectric materials, which generate electrical charges upon mechanical deformation, hold great potential for harvesting energy from ambient sources, including human motion, vibrations, and sound waves [20, 21, 22, 23, 24]. Recently, flexible piezoelectric devices have seen significant development in the field of wearable sensing. For example, ultra‐thin stretchable devices based on ferroelectric electrets are now capable of effectively monitoring facial muscle activity [25]. However, their reliance on non‑biodegradable polymer substrates prevents full system biodegradability, thereby limiting their applicability in transient, eco‑friendly, or implantable electronic systems. In parallel, biodegradable piezoelectric materials derived from natural sources, such as collagen [26], chitosan [27], fish swim bladder [28], and γ‐glycine [29], have been explored. Nevertheless, these materials currently suffer from substantially lower piezoelectric coefficients than conventional ferroelectric ceramics, an intrinsic limitation that severely constrains their electromechanical conversion efficiency and practical effectiveness in mechanical sensing applications.

**FIGURE 1:**
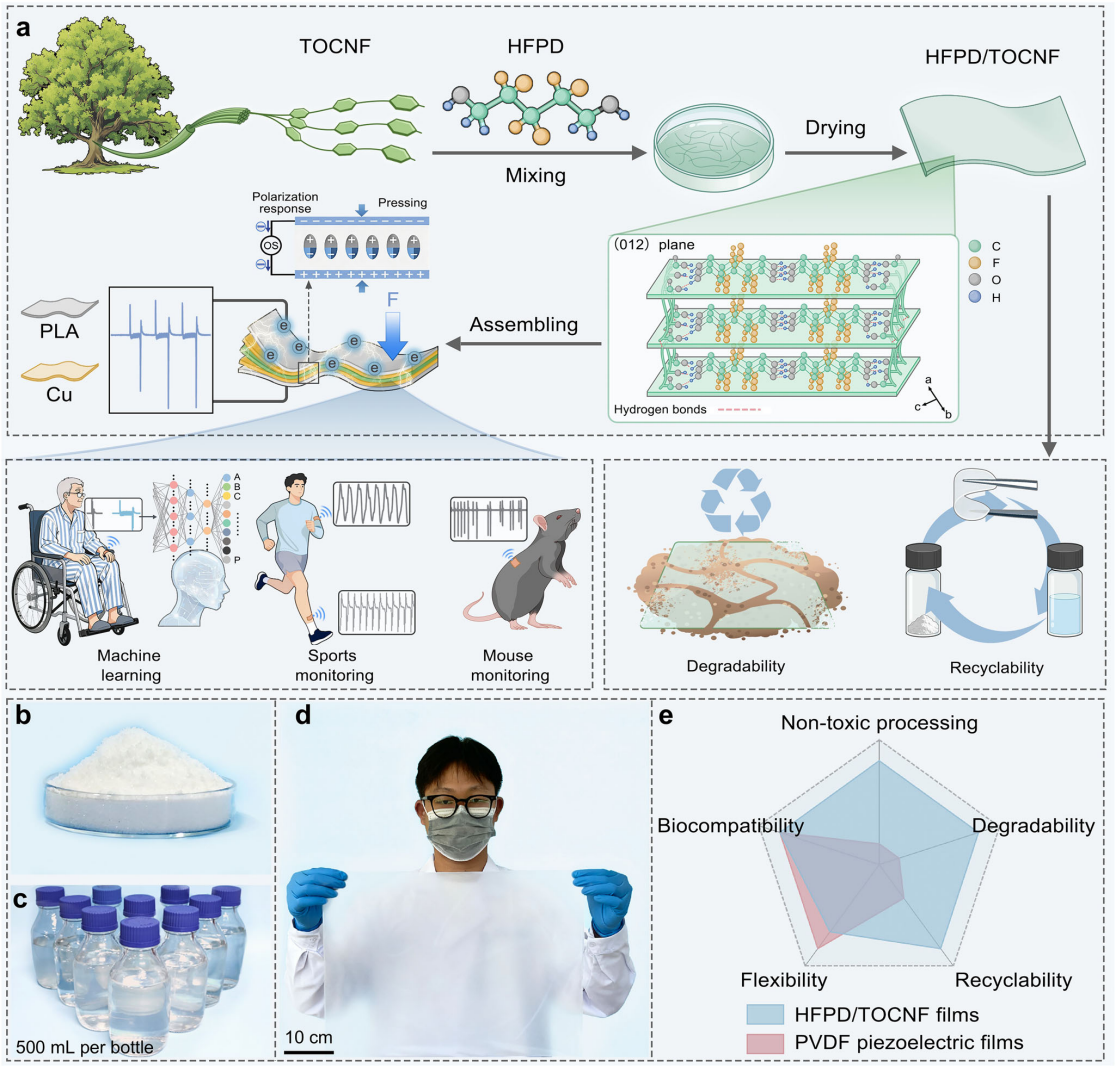
Preparation and characteristics of HFPD/TOCNF piezoelectric composite films. (a) Schematic of the design strategy: nanocellulose extracted from wood biomass is composited with HFPD molecular crystals to construct a high‐performance nanogenerator for sustainable sensing. (b) Photograph of the HFPD molecular crystals. (c) HFPD/TOCNF mixed solution. (d) A large‐scale sheet of HFPD/TOCNF composite film (50 cm × 40 cm). (e) Radar plot comparing the integrated performance of the HFPD/TOCNF film with a representative PVDF piezoelectric film.

In this work, we present a high‐performance, fully biodegradable piezoelectric composite that bridges the gap between functional performance and environmental sustainability. The composite combines bamboo‐derived cellulose nanofibers with the piezoelectric molecular crystal 2,2,3,3,4,4‐hexafluoropentane‐1,5‐diol (HFPD) [30], using a simple solvent evaporation process. Unlike pure HFPD, which is a brittle crystal that cannot be directly processed into flexible devices, our HFPD/TOCNF composite successfully transforms this high‐performance molecular ferroelectric into a flexible, recyclable, and fully biodegradable practical piezoelectric material. Cellulose is renewable, biodegradable [31, 32], and intrinsically piezoelectric due to its non‐centrosymmetric crystalline structure [33, 34, 35, 36], but its low piezoelectric coefficient (*d*_33_ ≈ 0.4 pC·N^−1^) limits its practical use [37]. By introducing carboxylate groups through TEMPO‐mediated oxidation, we enhance the interfacial interactions between cellulose and HFPD, enabling the high piezoelectricity of HFPD (*d*_33_ ≈ 138 pC·N^−1^) to be effectively transferred into the composite to achieve a high piezoelectric coefficient (14.0 pC·N^−1^). This design facilitates the formation of a hierarchically ordered structure stabilized by multiscale hydrogen bonding, which significantly improves the electromechanical performance. The resulting composite film serves as a self‐powered piezoelectric device capable of efficiently harvesting biomechanical energy and monitoring human motion and physiological signals with high sensitivity (Figure 1a). Importantly, this composite is processed with water‐assisted techniques, ensuring full recyclability and material recovery without compromising functionality, and completely eliminating the use of toxic organic solvents. Compared to traditional piezoelectric materials (e.g., PZT), HFPD/TOCNF offers superior biodegradability, enhanced recyclability, reusability, flexibility, and fatigue resistance (100 000 bending cycles). These advantages make HFPD/TOCNF composites a scalable and environmentally friendly alternative with broad potential for diverse applications.

## 2 Results and Discussion

### 2.1 Design Strategy of High‐Performance HFPD/TOCNF Composite Films

High‐performance HFPD/TOCNF composite films were rationally designed by integrating two environmentally benign components: piezoelectric molecular crystals of HFPD and TEMPO‐oxidized cellulose nanofibers. As illustrated in Figure 1b, HFPD exhibits a non‐centrosymmetric molecular structure that underpins its intrinsic piezoelectricity. Uniform mixing of HFPD crystals with TOCNF in aqueous media yielded a homogeneous dispersion (Figure 1c), which subsequently underwent solvent evaporation‐induced crystallization to form mechanically robust composite films. At the molecular level, abundant hydrogen‐bonding interactions were established between HFPD molecules and the carboxylate and hydroxyl groups of TOCNF, resulting in a hierarchical, multilevel hydrogen‐bonding network. This interfacial architecture effectively reinforced the composite structure while facilitating efficient stress transfer and polarization alignment, thereby providing a structural basis for enhanced piezoelectric performance. Importantly, the fabrication process is inherently scalable. Large‐area composite films with dimensions up to 50 cm × 40 cm were readily produced without compromising structural integrity or uniformity (Figure 1d). Moreover, the composites are fully compatible with water‐assisted processing and recycling, enabling material recovery and reprocessing with minimal energy input and without the use of toxic organic solvents (Figure 1e and Table S1). Collectively, these features demonstrate that the HFPD/TOCNF system constitutes a scalable and sustainable alternative to conventional non‐degradable piezoelectric polymers and ceramic‐based materials.

### 2.2 Structural Characterization of TOCNF/HFPD Composite Films

**FIGURE 2:**
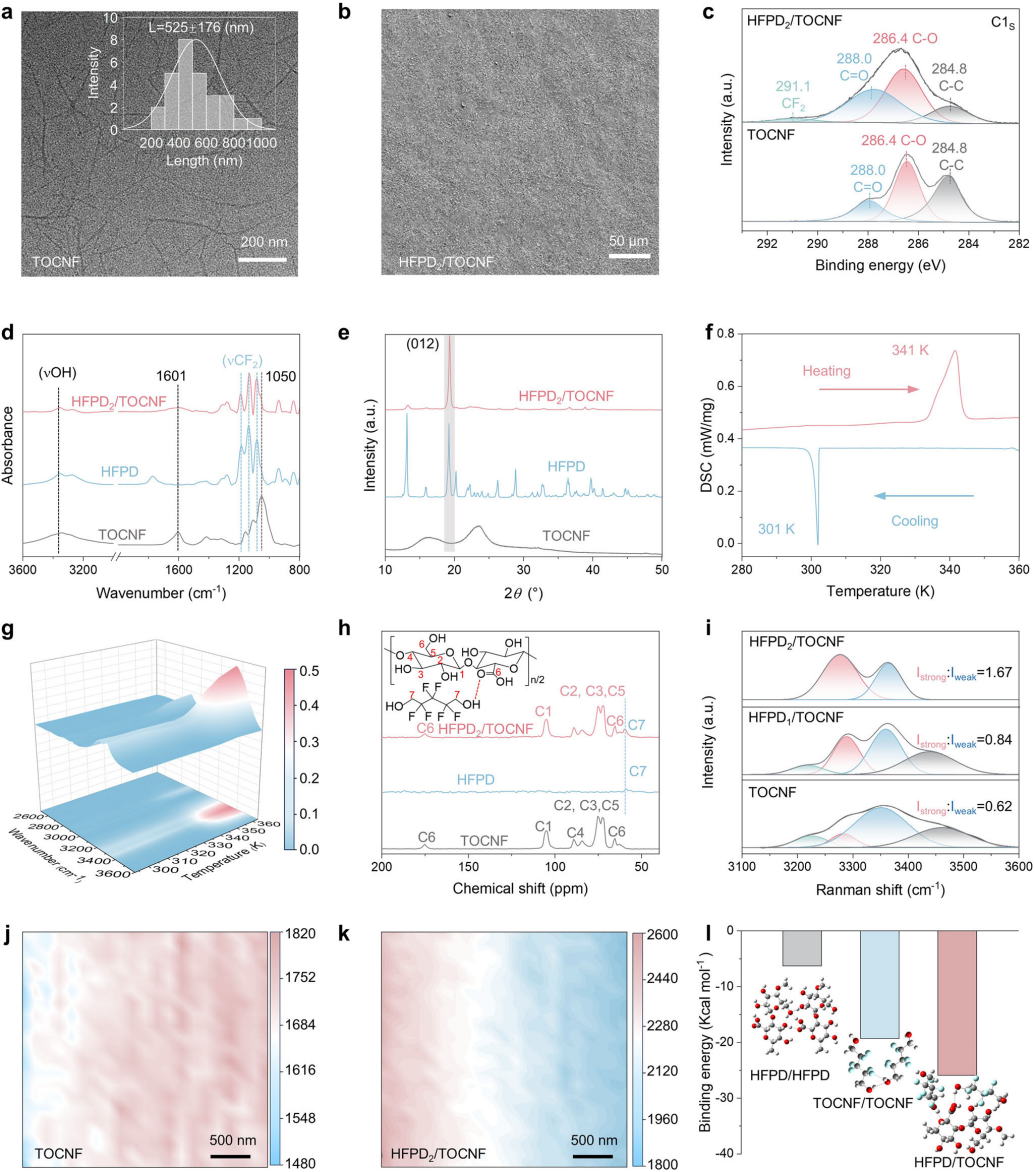
Structural characterization and interactions within the HFPD/TOCNF composite films. (a) TEM image of TOCNF. (b) SEM image of HFPD/TOCNF (2:1) film. (c) XPS spectra of TOCNF and HFPD/TOCNF films. (d) IR spectra of TOCNF and HFPD/TOCNF films. (e) XRD patterns of TOCNF, pure HFPD, and HFPD/TOCNF composite films. (f) DSC curves of HFPD/TOCNF. (g) VT‐FTIR spectra of HFPD/TOCNF from 20°C to 90°C. (h) Solid‐state ^13^C NMR spectra of TOCNF and HFPD/TOCNF. (i) Raman spectra showing O─H stretching vibrations in the HFPD/TOCNF film. 2D Raman mapping images of (j) TOCNF and (k) HFPD/TOCNF films. (l) DFT‑calculated binding energy of HFPD/HFPD, TOCNF/TOCNF, and HFPD/TOCNF.

To elucidate the structural basis underlying the enhanced piezoelectric performance of the HFPD/TOCNF composites, their nanoscale morphology and interfacial architecture were systematically investigated. Transmission electron microscopy (TEM) revealed that the TOCNF exhibited an average length of 525 ± 176 nm and a width of 10.2 ± 3.1 nm, resulting in a high aspect ratio (Figure 2a). Such elongated nanofibers favor the formation of an interconnected fibrous network within the composite films, which is conducive to efficient stress transfer and signal propagation under mechanical deformation. The surface morphology of the films was further examined by scanning electron microscopy (SEM). Pristine TOCNF films displayed smooth and compact surfaces with a uniform texture (Figure S1a). In contrast, incorporation of HFPD induced the formation of discernible crystalline domains, leading to increased surface roughness in the composite films (Figure 2b and Figure S1b). Notably, at HFPD/TOCNF mass ratios exceeding 2:1, the films maintained good structural uniformity without apparent phase separation (Figure S1c,d). Elemental mapping analysis confirmed the homogeneous distribution of fluorine (F) throughout the HFPD/TOCNF (2:1) composite films (Figure S1e), indicating uniform incorporation of HFPD within the TOCNF matrix. X‐ray photoelectron spectroscopy (XPS) further substantiated the successful integration of HFPD. Compared to pure TOCNF, which showed no fluorine signal, the full survey spectrum of HFPD/TOCNF films exhibited a distinct F 1s peak at 688.3 eV (Figure S2) [38]. High‐resolution C 1s spectra of pure TOCNF were deconvoluted into peaks corresponding to C─C/C─H (284.8 eV), C─O (286.4 eV), and O─C─O (288.0 eV) [39]. Upon incorporation of HFPD, an additional C 1s component at 291.1 eV, assigned to ─CF_2_ moieties [40], was clearly observed in the composite films (Figure 2c).

Surface functional groups and molecular interactions within the composite films were further examined by attenuated total reflectance Fourier‐transform infrared spectroscopy (ATR‐FTIR) (Figure 2d). A broad O─H stretching band centered at 3423 cm^−1^, attributed to surface hydroxyl groups [41], dominated the spectra. Upon incorporation of HFPD, new absorption peaks appeared at 1187, 1130, and 1086 cm^−1^, corresponding to the stretching vibrations of ─CF_2_ groups [42, 43], aligning with the XPS results. The asymmetric distribution of C─F bonds in these groups generates a non‐zero molecular dipole moment, which underpins the composite's piezoelectric properties [44]. In addition, characteristic vibrations of nanocellulose were clearly preserved. The peak at 1601 cm^−1^ is assigned to the stretching vibration of carboxyl (C═O) groups associated with sodium carboxylate (─COONa) [45], while the peak at 1050 cm^−1^ corresponds to the C─O─C stretching vibration of the cellulose backbone [46, 47], confirming the structural integrity of TOCNF after composite formation. X‐ray diffraction (XRD) analysis of the HFPD/TOCNF (2:1) film further revealed a pronounced diffraction peak at 19.1°, which can be indexed to the (012) plane of crystalline HFPD (Figure 2e). Crystallographically, this plane consists of 2D hydrogen‐bonded layers, in which HFPD molecules are interconnected via O─H···O hydrogen bonds [30]. Such well‐defined molecular ordering and continuous crystallization are expected to facilitate dipole alignment under mechanical stress, thereby contributing to the enhanced piezoelectric response of the composite films.

To elucidate the thermal behavior and phase evolution of the composite films, differential scanning calorimetry (DSC) was performed on HFPD_2_/TOCNF (the ratio of HFPD to TOCNF is 2:1) composites with varying compositions (Figure 2f). The DSC traces reveal that HFPD undergoes reversible melting and crystallization within the TOCNF matrix. Specifically, the composite films exhibit a distinct endothermic peak at approximately 335–350 K during heating, corresponding to the melting temperature (*T*_m_) of HFPD, and an exothermic peak between 290 and 312 K upon cooling, associated with crystallization (*T*_c_). The reversible melting‐crystallization behavior indicates that HFPD molecules retain sufficient mobility to reorganize into ordered crystalline domains within the nanocellulose framework, which is essential for sustaining a stable polar phase. With increasing HFPD content, both *T*_m_ and *T*_c_ gradually shift to higher temperatures, suggesting that the crystallization capacity has been enhanced and crystalline perfection improved due to strengthened intermolecular interactions. At higher HFPD loadings (mass ratios of 3:1 and 4:1), however, the DSC curves display a pronounced double‐peak feature (Figure S3a,b), indicative of the coexistence of multiple crystalline populations. This behavior can be attributed to incomplete confinement of HFPD within the TOCNF network, allowing a fraction of HFPD chains to crystallize outside the nanocellulose scaffold. These unconstrained crystalline domains are expected to exhibit more random molecular orientations, which may partially offset dipole alignment and thus diminish the overall piezoelectric response.

To further probe the temperature‐dependent evolution of hydrogen bonding, variable‐temperature infrared spectrometry (VT‐FTIR) measurements were carried out. For the TOCNF film, the broad O─H stretching band in the 3200–3400 cm^−1^ region gradually weakened and narrowed with increasing temperature (Figure S4a), reflecting the progressive disruption of hydrogen bonds due to water desorption and thermal disordering. Concurrently, in the 1500–1700 cm^−1^ region, a shift toward higher wavenumbers was observed (Figure S4b), which can be attributed to H─O─H bending vibrations of water molecules and asymmetric stretching of carboxylate groups, further confirming the loss of bound water and weakening of hydrogen‐bond networks upon heating. In contrast, the HFPD/TOCNF (2:1) composite film exhibited two well‐resolved O─H stretching bands centered at approximately 3350 and 3250 cm^−1^ (Figure 2g). The higher‐wavenumber band (∼3350 cm^−1^) is associated with relatively weak and disordered hydrogen bonds predominantly within the TOCNF matrix, whereas the lower‐wavenumber band (∼3250 cm^−1^) originates from strong, highly ordered hydrogen bonds within the 2D HFPD molecular layers, which play a critical role in the enhanced piezoelectric response. Upon heating, the ∼3250 cm^−1^ band progressively weakened and nearly vanished above *T*_m_, while the ∼3350 cm^−1^ band intensified, signifying an order‐to‐disorder transition of the HFPD hydrogen‐bonded network, in agreement with the DSC results (Figure S4c). Correspondingly, in the 1500–1700 cm^−1^ region (Figure S4d), the HFPD/TOCNF composite initially showed no distinct absorption features, indicating that the strong, ordered hydrogen‐bond interactions constrained the vibrational freedom of carboxyl‐related groups. Once the temperature exceeded *T*_m_, these ordered domains were disrupted, liberating HFPD molecules, TOCNF chains, carboxyl groups, and associated water molecules. The resulting increase and broadening of the band reflect the formation of numerous new, dynamically fluctuating hydrogen bonds.

To further elucidate the molecular‐level interactions between HFPD and TOCNF, solid‐state ^13^C NMR spectroscopy was conducted on both pristine TOCNF and HFPD/TOCNF composite films (Figure 2h). In the TOCNF spectrum, resonances at 175.1 ppm, 105.2 ppm, 84–89 ppm, 72–75 ppm, and 63–66 ppm were assigned to the C6 carboxylate groups introduced by TEMPO oxidation, the C1 position, C4, C2/C3/C5, and C6 carbons of the cellulose backbone, respectively [48, 49]. Upon incorporation of HFPD, the composite film exhibited a pronounced new resonance at approximately 60 ppm, characteristic of HFPD molecules. Concurrently, a slight decrease in the intensity of the C6 signal and the C4 subpeak of TOCNF was observed, indicative of changes in the local chemical environment of these carbons. Such spectral variations are consistent with the formation of intermolecular hydrogen bonds between HFPD and TOCNF [50].

Raman spectroscopy provided complementary evidence for enhanced hydrogen bonding (Figure 2i). The composite films displayed a strong hydrogen‐bond‐associated band at 3270 cm^−1^ and a weaker band at cm^−1^. Notably, the intensity ratio of strong to weak hydrogen bonds (*I*_s_:*I*_w_) increased from 0.62 in pristine TOCNF to 1.67 in HFPD/TOCNF (2:1), reflecting a substantial enhancement in hydrogen‐bond strength and density following HFPD incorporation [51]. Consistently, the overall hydroxyl‐related Raman intensity increased in the composite films (Figure S5), and 2D Raman mapping revealed a dense hydrogen‐bonding network within the 3200–3400 cm^−1^ region (Figure 2j,k). Furthermore, density functional theory (DFT) calculations were performed to quantify the intermolecular interactions between HFPD and TOCNF (Figure 2l). The calculated binding energy (E binding), where more negative values indicate stronger interactions, was −25.89 kcal·mol^−1^ for the HFPD/TOCNF pair. This value is significantly lower than those calculated for HFPD/HFPD (−6.28 kcal·mol^−1^) and TOCNF/TOCNF (−19.28 kcal·mol^−1^), suggesting a stronger and more stable interaction between the components in the HFPD/TOCNF composite, enabling the material to maintain a robust piezoelectric response under mechanical deformation.

### 2.3 Mechanical and Piezoelectric Properties of HFPD/TOCNF Composite Films

**FIGURE 3:**
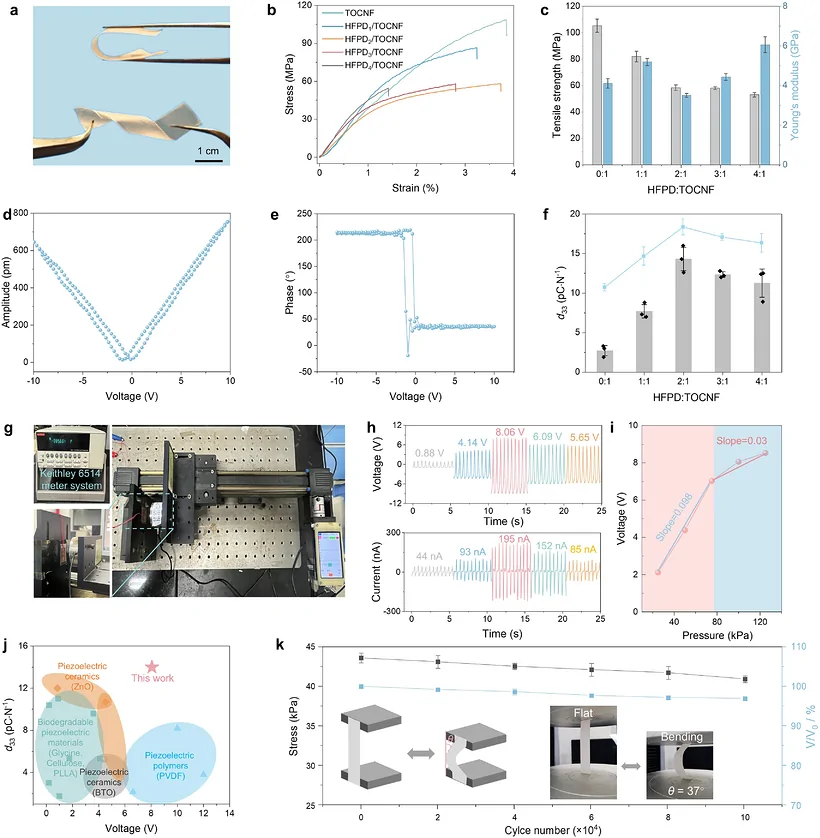
Mechanical and piezoelectric performance of HFPD/TOCNF composite films. (a) Flexibility of HFPD/TOCNF composite film. (b) Stress–strain curves for films with different HFPD/TOCNF ratios. (c) Young's modulus and tensile strength for films with different HFPD/TOCNF ratios. (d) Amplitude‐voltage curve of HFPD/TOCNF. (e) Phase‐voltage curves of HFPD/TOCNF. (f) *d*_33_ of composite films with various HFPD/TOCNF ratios. (g) Schematic of the HFPD/TOCNF PENG structure and measurement setup. (h) *V*_OC_ and *I*_SC_ of composite films with various HFPD/TOCNF ratios. (i) Voltage response to a wide range of pressures. (j) Performance comparison of the HFPD/TOCNF film with reported piezoelectric materials in terms of *d*_33_ and output voltage. (k) Changes in the mechanical and piezoelectric properties of HFPD/TOCNF piezoelectric films during a bending process of 100 000 cycles.

To assess the mechanical behavior of the composite films, tensile stress–strain responses were systematically evaluated as a function of the HFPD‐to‐TOCNF mass ratio. At an HFPD/TOCNF ratio of 2:1, the composite films exhibited excellent flexibility, maintaining structural integrity under repeated folding and twisting (Figure 3a). Quantitative tensile testing revealed that pristine TOCNF films possessed a tensile strength of 105.3 ± 4.9 MPa with a limited elongation at break of 3.8 ± 0.1% (Figure 3b). With increasing HFPD incorporation, both tensile strength and elongation at break gradually decreased. Concurrently, Young's modulus increased sharply with rising HFPD content (Figure 3c), indicating a progressive transition from a ductile to a more brittle mechanical response. This trend was further corroborated by the reduction in fracture energy at higher HFPD loadings (Figure S6), reflecting increased rigidity and reduced energy dissipation during fracture. These results indicate that while HFPD integration reinforces the stiffness of the TOCNF matrix, excessive HFPD introduces structural constraints that compromise flexibility. Importantly, this behavior highlights the tunable nature of the composite system, in which mechanical robustness and deformability can be balanced by adjusting the HFPD content.

The intrinsic piezoelectric behavior of the composite films was investigated using piezoresponse force microscopy (PFM). Both TOCNF and HFPD/TOCNF films exhibited characteristic butterfly‐shaped amplitude loops (Figure 3d and Figure S7c) and 180° phase‐switching hysteresis loops (Figure 3e and Figure S7d), confirming their piezoelectric nature. Under a 10 V AC excitation, phase images revealed clear phase contrast associated with electromechanical coupling, while amplitude images visualized the spatial distribution of the piezoresponse through brightness variations (Figure S7a,b,e,f). Notably, the HFPD/TOCNF composite displayed more symmetric butterfly loops and well‐defined phase reversal behavior compared to pristine TOCNF, indicative of enhanced polarization switching. The piezoelectric coefficient measured by PFM reflects the local nanoscale piezoelectric response, whilst the quasi‐static *d*_33_ serves as a macroscopic benchmark for device performance. The effective piezoelectric coefficient extracted from PFM measurements reached 64.79 pm·V^−1^, substantially exceeding that of the TOCNF film. To quantitatively evaluate macroscopic energy conversion efficiency, the piezoelectric charge coefficient (*d*_33_) was further measured. Pure TOCNF films exhibited a low *d*_33_ value of 2.55 pC·N^−1^, whereas incorporation of HFPD resulted in a pronounced enhancement (Figure 3f). At an optimal HFPD/TOCNF ratio of 2:1, the composite achieved a maximum *d*_33_ of 14.0 ± 1.5 pC·N^−1^, demonstrating effective transfer of the high piezoelectric activity of HFPD into the nanocellulose matrix.

To evaluate practical energy‐harvesting performance, the HFPD/TOCNF composite film was assembled into a sandwich‐structured piezoelectric nanogenerator (PENG), consisting of copper electrodes on both sides of the composite and encapsulated with polylactic acid (PLA) to prevent environmental charge interference [52]. The device configuration and testing setup are illustrated in Figure 3g, with the working mechanism depicted in Figure S8. Under open‐circuit conditions, randomly oriented dipoles produce no net polarization. Upon mechanical compression, dipole alignment induces charge polarization and drives electron flow between the electrodes, while stress release generates a reverse current [53]. Under a compressive stress of 100 kPa, the HFPD/TOCNF‐based PENG delivered an open‐circuit voltage (*V*_OC_) of 8.06 V and a short‐circuit current (*I*_SC_) of 195 nA (Figure 3h), corresponding to a 91.6% increase in *V*_OC_ and an approximately fourfold enhancement in *I*_SC_ compared to pristine TOCNF. The maximum output power and power density reached 1.52 ± 0.04 µW and 3.80 ± 0.11 mW·m^−2^, respectively (Figure S9a), confirming the superior energy‐harvesting capability of the composite. Notably, the piezoelectric output exhibited a non‐linear dependence on HFPD content, closely mirroring the trend observed for *d*_33_ (Figure 3f). While the 2:1 ratio yielded optimal performance, further increases in HFPD content led to a 32% reduction in *V*_OC_, likely due to phase separation under high HFPD loading (as evidenced by the double‑peak in DSC), which causes part of the HFPD to crystallize freely outside the TOCNF network with random orientation, thereby disrupting the ordered polar structure required for piezoelectricity. The polarization of crystals with different orientations cancels each other out, ultimately leading to a decrease in piezoelectric performance. At the same time, compromised mechanical integrity and increased brittleness may cause microstructural damage under repeated loading, further contributing to the reduction in piezoelectric output. The piezoelectric response also showed a strong dependence on applied stress. For the HFPD/TOCNF (2:1) composite, both *V*_OC_ and *I*_SC_ increased proportionally with increasing impact pressure (Figure S9b,c). The device exhibited a sensitivity of 0.098 kPa^−1^ below 75 kPa, which decreased to 0.030 kPa^−1^ in the 75–125 kPa range (Figure 3i), reflecting stress‐dependent deformation behavior and mechanical stiffening at higher compressive stresses. The HFPD/TOCNF composite demonstrates competitive or superior performance compared with previously reported piezoelectric films [29, 54, 55, 56, 57, 58, 59, 60, 61, 62, 63, 64, 65, 66] (Figure 3j and Table S3), indicating strong potential for sustainable energy harvesting and sensing. Notably, it exhibits excellent durability, retaining 94% of its initial mechanical strength and 97% of its piezoelectric performance after 100 000 bending cycles (Figure 3k).

### 2.4 HFPD/TOCNF PENG for Energy Harvesting and Biomonitoring

To demonstrate the feasibility of the HFPD/TOCNF piezoelectric nanogenerator (PENG) for practical energy harvesting, its capability to charge commercial capacitors through a rectifier bridge was systematically evaluated. Under cyclic mechanical excitation, the charging voltage across capacitors with capacitances ranging from 1.0 to 10 µF increased nearly linearly during the initial charging stage and gradually reached saturation after approximately 400 s. Final voltages of 2.57, 1.50, 0.86, 0.70, and 0.55 V were obtained for 1.0, 2.2, 4.7, 6.8, and 10 µF capacitors, respectively (Figure S10), confirming efficient mechanical‐to‐electrical energy conversion and stable power delivery. These results demonstrate the potential of the HFPD/TOCNF PENG as a sustainable power source for low‐power wearable electronics.

**FIGURE 4:**
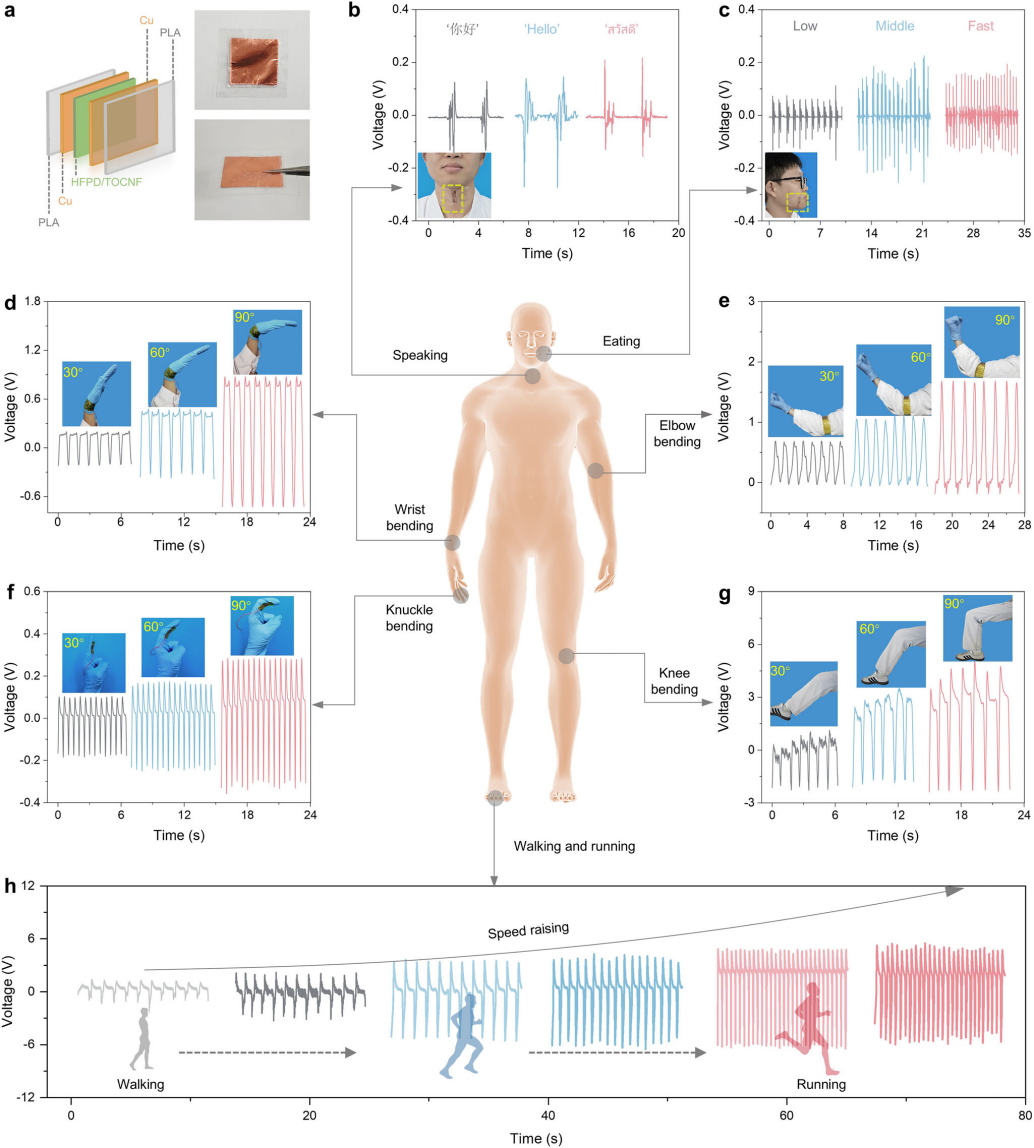
Applications of HFPD/TOCNF PENG for wearable sensors. (a) Schematic diagram of HFPD/TOCNF piezoelectric sensor. HFPD/TOCNF piezoelectric sensor for monitoring (b) speech and (c) eating. Joint motion detection and gait analysis through bending signals from the (d) wrist, (e) elbow, (f) knuckle, (g) knee, and (h) gait frequency variations under the foot sole.

Cellulose‐based materials have recently emerged as promising candidates for wearable and implantable biomedical applications [67]. Benefiting from its flexibility and high piezoelectric sensitivity, the HFPD/TOCNF composite film was further integrated into a flexible, biocompatible PENG by sandwiching the active layer between copper electrodes and encapsulating it with polylactic acid (PLA). The resulting device exhibited excellent conformability to human skin and high responsiveness to biomechanical deformation, enabling its application as a wearable sensor for both subtle physiological signals and large‐scale joint movements (Figure 4a). When attached to the throat, the sensor successfully captured weak biomechanical signals associated with muscle contraction and skin deformation during speech. Distinct voltage signatures were recorded for spoken words in different languages, including “你好,” “Hello,” and “สวัสดี” (Figure 4b), highlighting the device's sensitivity and signal reproducibility. In addition, the sensor effectively monitored chewing motions when placed near the mouth (Figure 4c), offering a non‐invasive approach to assess oral activity patterns relevant to digestive health and temporomandibular joint function. The HFPD/TOCNF PENG also demonstrated reliable performance in monitoring multi‐joint human motions. For wrist movements, peak output voltages of approximately 0.17, 0.45, and 0.86 V were generated at flexion angles of 30°, 60°, and 90°, respectively (Figure 4d). Similar angle‐dependent responses were observed for elbow flexion, with voltages of ∼0.69, ∼1.05, and ∼1.69 V at corresponding angles (Figure 4e), as well as for knuckle and knee motions (Figure 4f,g). Moreover, the device clearly distinguished between fast (∼3 Hz) and slow (∼1 Hz) motion frequencies (Figure 4h), achieving a temporal resolution of ∼0.33 s. These results underscore the suitability of the HFPD/TOCNF sensor for continuous, real‐time motion monitoring in wearable healthcare and human‐machine interface applications.

**FIGURE 5:**
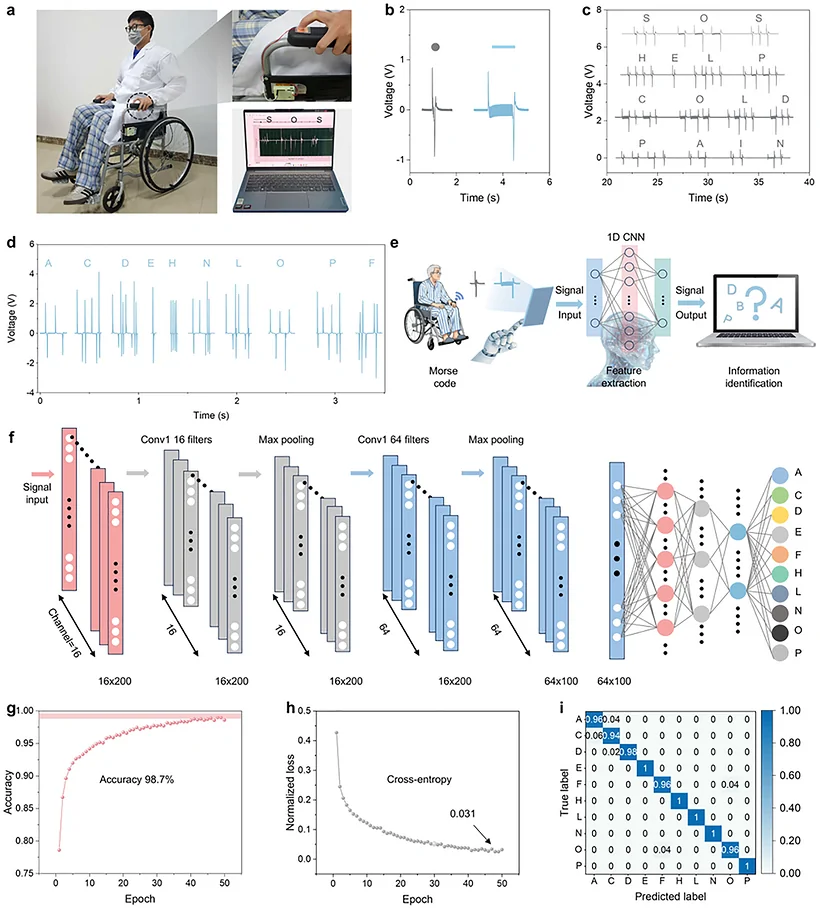
IoT‐integrated HFPD/TOCNF sensors for remote health monitoring and assisted communication. (a) Application in Morse code communication. (b) Piezoelectric signals of dots ‘·’ and dashes ‘‐’. (c) Voltage outputs corresponding to four example words: ‘SOS’, ‘COLD’, ‘PAIN’, and ‘HELP’. (d) Signal patterns for letters A, C, D, E, H, N, L, O, P, and F. (e) Schematic of deep learning‐assisted Morse code recognition. (f) 1D CNN architecture. (g) Prediction accuracy at different iteration counts. (h) Normalized loss of the cross‐entropy function. (i) Confusion matrix for recognizing letters.

Building on its reliable sensing capability, we further explored the integration of the HFPD/TOCNF sensor with Internet of Things (IoT) platforms for assisted communication and remote health monitoring. In a proof‐of‐concept demonstration, users wearing the device were able to convey physiological states or discomfort (e.g., “COLD” or “PAIN”) to clinicians via voice‐triggered sensing, with data transmitted wirelessly through Bluetooth or WiFi (Figure 5a). For nonverbal or speech‐impaired users, Morse code was implemented as an alternative communication modality. In this scheme, short and long sensor presses generated distinct voltage signals corresponding to dots (·) and dashes (−), respectively (Figure 5b). Using this approach, representative emergency messages, including “SOS,” “COLD,” “PAIN,” and “HELP,” were successfully encoded and transmitted (Figure 5c). Full dynamic demonstrations are provided in Videos S1 (“SOS”), S2 (“COLD”), S3 (“PAIN”), and S4 (“HELP”), which collectively highlight the versatility of the HFPD/TOCNF PENG as a multifunctional human‐machine interface.

To establish a robust relationship between sensor output signals and encoded information, deep learning techniques were integrated with the HFPD/TOCNF sensing system. Characteristic piezoelectric signal features generated by rhythmic finger‐pressing motions were extracted and used to train an intelligent recognition framework. In this study, ten Morse code letters (“A”, “C”, “D”, “E”, “F”, “H”, “L”, “N”, “O” and “P”) were selected as representative classes (Figure 5d). The implementation workflow is outlined in Figure 5e. Signal data from volunteer finger presses were first collected and processed for feature extraction, followed by model training and automatic Morse code recognition using a 1D convolutional neural network (1D‐CNN). Due to its translation invariance and strong temporal feature‐learning capability, the 1D‐CNN served as the core classification model (Figure 5f). The network architecture consisted of two convolutional layers (16 and 64 filters), each followed by a 2 × 2 pooling layer, with performance monitored using a cross‐entropy loss function. As training progressed, classification accuracy increased steadily (Figure 5g), while the loss value decreased to 0.031 after 50 iterations (Figure 5h). To ensure robustness, 50 signal sequences were collected for each letter, yielding a total of 5 000 data points. Confusion matrix analysis revealed 100% classification accuracy for several letters (E, H, L, N, and O), with an overall accuracy of 98.7% (Figure 5i). This high recognition performance arises from the combination of the sensor's sensitivity to subtle biomechanical inputs and the deep learning model's ability to discriminate complex temporal patterns. Collectively, these results demonstrate that HFPD/TOCNF‐based PENGs, when coupled with intelligent algorithms, can enable efficient, real‐time assistive communication for individuals with limited mobility or speech impairments.

### 2.5 Biocompatibility and Biosensing of HFPD/TOCNF PENG

**FIGURE 6:**
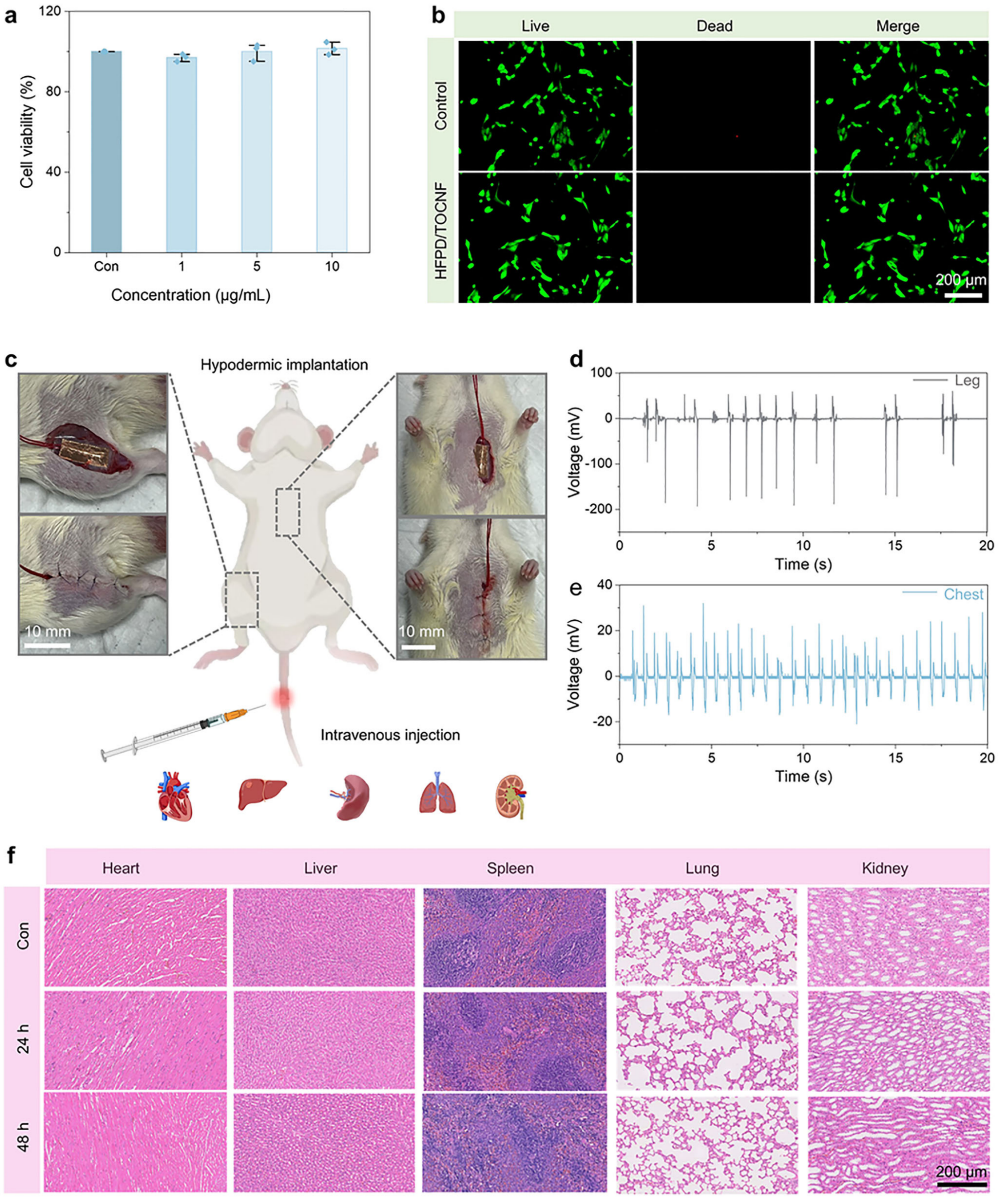
Biological properties of HFPD/TOCNF composite films. (a) Viability of NIH/3T3 cells cultured with different concentrations of HFPD/TOCNF. (b) Fluorescence images of NIH/3T3 cells after 24 h incubation with HFPD/TOCNF, stained with AM (green, live) and PI (red, dead). (c) Photographs of HFPD/TOCNF devices implanted in the thigh and chest, and intravenously injected into SD rats. (d) Piezoelectric output from the device implanted on the quadriceps femoris during gentle stretching. (e) Respiratory‐driven voltage signal from the device on the pectoralis major muscle. (f) H&E‐stained images of major organs from control (PBS‐injected) and HFPD/TOCNF‐treated rats at 24 and 48 h post‐injection.

The biocompatibility of the HFPD/TOCNF composite films was evaluated in vitro using mouse embryonic fibroblasts (NIH/3T3 cells). Cell viability was quantified by the Cell Counting Kit‐8 (CCK‐8) assay after incubation with culture media containing 1, 5, and 10 µg·mL^−1^ of HFPD/TOCNF. As shown in Figure 6a, the composite exhibited negligible cytotoxicity, maintaining cell viability above 100% even at the highest tested concentration (10 µg·mL^−1^). Consistently, live/dead staining using calcein‐AM and propidium iodide (PI) revealed predominantly green fluorescence, indicating a high proportion of viable cells. Moreover, cells cultured with HFPD/TOCNF displayed normal morphology and spreading behavior comparable to the control group (Figure 6b), confirming the excellent cytocompatibility of the composite films. Encouraged by the favorable in vitro results, the in vivo biocompatibility and functional performance of the HFPD/TOCNF PENG were further investigated. Miniaturized PENG devices (5 mm × 10 mm) fabricated from the composite films were subcutaneously implanted into the thigh and chest regions of rats (Figure 6c). When the hind limb was gently stretched at different frequencies, the device implanted near the quadriceps muscle generated stable electrical outputs exceeding 100 mV (Figure 6d and Video S5). Similarly, the PENG implanted on the pectoralis major muscle produced reproducible voltage signals of approximately 20 mV in response to respiratory movements (Figure 6e and Video S6). These results demonstrate that the HFPD/TOCNF PENG can effectively transduce subtle biomechanical deformations into electrical signals under physiological conditions.

To comprehensively assess systemic biosafety, an in vivo toxicity evaluation was conducted by intravenously administering the HFPD/TOCNF mixture (80 mg·kg^−1^) to mice via tail‐vein injection. Histological analysis of major organs, including the heart, liver, spleen, lung, and kidney, was performed at 24 and 48 h post‐injection. As shown in Figure 6f, no discernible pathological abnormalities were observed in any examined tissues compared with the control group. All organs maintained intact tissue architecture, with no evidence of acute injury, inflammatory infiltration, hemorrhage, or edema. Collectively, these in vitro and in vivo results demonstrate that the HFPD/TOCNF composite possesses excellent biocompatibility and does not induce acute systemic toxicity under the tested conditions. Coupled with its intrinsic piezoelectric activity and mechanical flexibility, the HFPD/TOCNF PENG represents a promising platform for implantable and wearable biomedical energy harvesting and sensing applications.

### 2.6 Recyclability and Biodegradability of HFPD/TOCNF PENG

**FIGURE 7:**
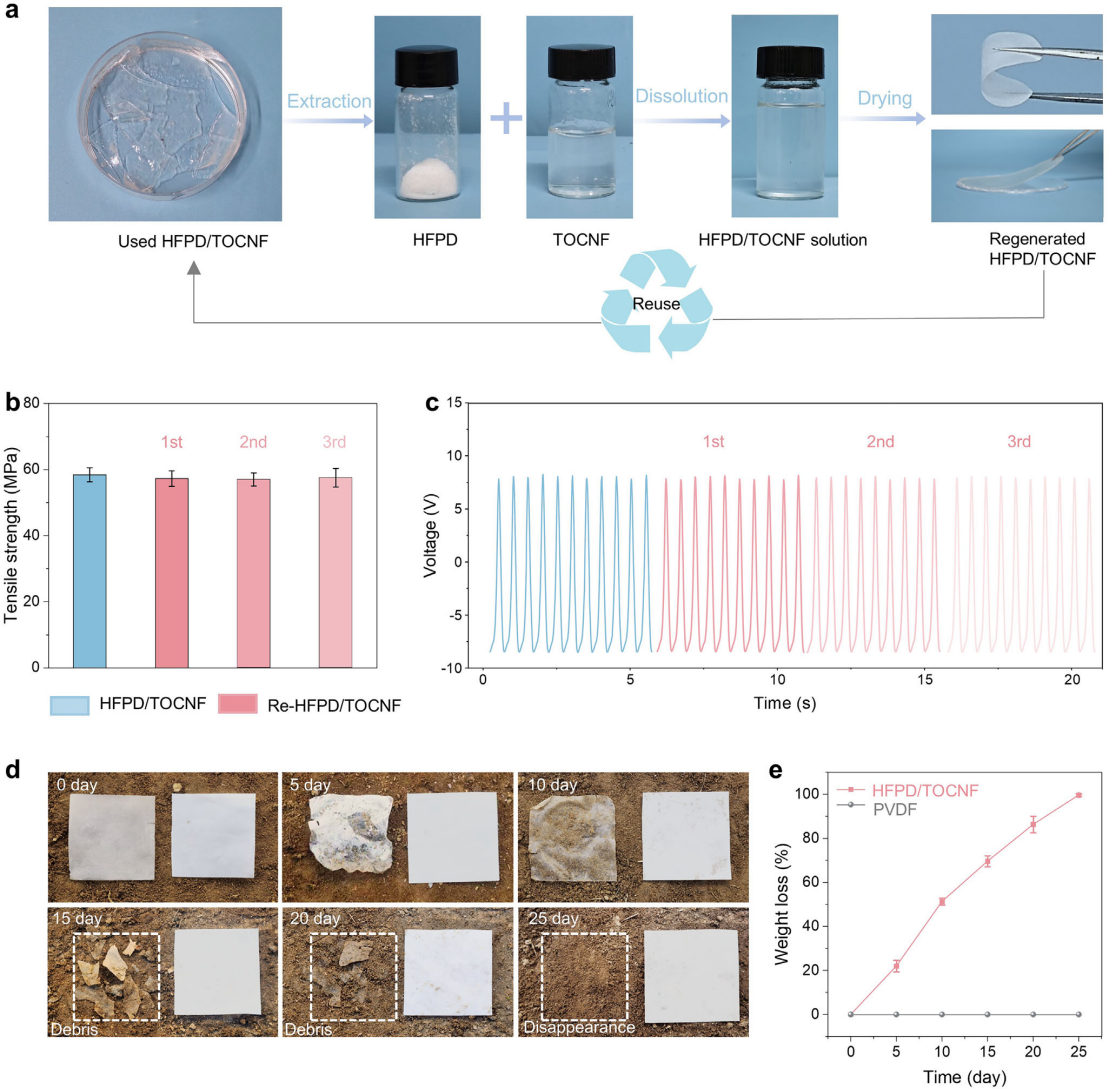
Recycling capability and soil degradation of HFPD/TOCNF composite materials. (a) Schematic of the film recycling process. (b) Tensile strength of the films before and after recycling. (c) *V*_OC_ output of the recycled PENGs. (d) Degradation test of HFPD/TOCNF and commercial PVDF films in natural soil. (e) Corresponding weight loss of HFPD/TOCNF composite films and commercial PVDF within 25 days.

Currently, most materials used in PENGs are derived from non‐degradable ceramics or petroleum‐based polymers, whose poor recyclability presents additional challenges to achieving carbon‐neutral goals. In contrast, recycling and reusing bio‐based resources is critical for reducing carbon footprints and accelerating the transition from fossil‐derived materials to renewable, sustainable alternatives [68]. In this regard, the HFPD/TOCNF composite offers a distinct advantage owing to the water‐dispersible nature of HFPD and the hydrophilic nanocellulose network. When immersed in water, HFPD molecules dissolve into the water phase, and the TOCNF network disassembles into a stable aqueous dispersion, enabling effective separation and recovery of the composite components; the film completely disintegrates within 6 h. The recyclability of the HFPD/TOCNF film was verified using the simplified regeneration process illustrated in Figure 7a. Briefly, the used composite film was immersed in water to induce disintegration, followed by vacuum filtration to recover crystalline HFPD as a white powder. The filtrate contains well‑dispersed TOCNF, which can also be recovered by centrifugation or re‑filtration. The recovered HFPD was subsequently redissolved, reintroduced into a fresh TOCNF suspension, and dried to form a regenerated composite film (denoted as Re‐HFPD/TOCNF). Remarkably, systematic evaluation of the regenerated films revealed that their mechanical properties and piezoelectric performance were largely preserved even after four times recycling‐regeneration cycles. As shown in Figure 7b,c, the tensile strength, fracture behavior, and electrical output of Re‐HFPD/TOCNF remained highly comparable to those of the pristine composite film. This closed‐loop regeneration capability offers two notable advantages: it minimizes dependence on virgin raw materials, thereby reducing resource consumption and waste generation, and it conforms to circular economy principles by enabling repeated reuse without performance degradation. Such recyclability represents a meaningful advance for sustainable piezoelectric devices in flexible electronics, wearable sensors, and transient functional systems.

In addition to recyclability, the HFPD/TOCNF composite films exhibit pronounced biodegradability. Outdoor soil burial tests were conducted over a 25‐day period (December 5–30), with commercial PVDF films used as non‐degradable controls (Figure 7d). Throughout the test duration, PVDF films retained their original morphology, showing no visible signs of degradation. In contrast, the HFPD/TOCNF films began to fracture within 5 days and underwent complete structural disintegration by Day 25. Gravimetric analysis further confirmed the biodegradation behavior. As shown in Figure 7e, the HFPD/TOCNF films exhibited nearly complete mass loss (∼100%) after 25 days of soil burial, whereas PVDF films displayed negligible mass change over the same period. The rapid degradation of HFPD/TOCNF films can be attributed to the susceptibility of cellulose‐based networks to microbial enzymatic hydrolysis under natural soil conditions [69]. A direct comparison of our HFPD/TOCNF composite with representative cellulose‐based piezoelectric materials is provided in Table S2, which summarizes key performance indicators including piezoelectric coefficient, output voltage, poling requirement, biodegradability, and recyclability. Together, these results demonstrate that the HFPD/TOCNF composite combines high‐performance piezoelectric functionality with full environmental degradability, offering a sustainable alternative to conventional PVDF‐based PENGs.

## 3 Conclusion

In summary, we have developed a high‐performance PENG based on a molecularly engineered HFPD/TOCNF composite. Through multiscale interfacial hydrogen bonding, the material achieves a hierarchically ordered structure that yields a high piezoelectric coefficient (14.0 pC·N^−1^), enabling efficient mechanical‐to‐electrical energy conversion. The device demonstrates exceptional sensitivity for real‐time monitoring of diverse human physiological signals and joint movements. By integrating deep learning‐assisted recognition, it further serves as an accurate and intuitive communication interface for users with speech impairments. The composite exhibits excellent biocompatibility and successfully functions as an implantable sensor for in vivo motion and respiration monitoring. Crucially, this material system transcends the conventional performance‐sustainability trade‐off. It is fully recyclable in water without performance loss and undergoes excellent biodegradation in soil within 25 days. This work establishes a scalable and eco‐conscious design paradigm, harmonizing high electromechanical performance with full lifecycle sustainability. It provides a foundational strategy for developing next‐generation green electronics, from wearable and implantable biomedical sensors to environmentally aligned human‐machine interfaces, paving a tangible path toward a sustainable technological future.

## 4 Experimental Section

### 4.1 Material Preparation

TEMPO‐oxidized cellulose nanofibers (TOCNF) were purchased from Tianjin Woodelf Biotechnology Co., Ltd. The as‐received aqueous dispersion had a cellulose concentration of 1.06 wt.% and a carboxylate content of 2.0 ± 0.2 mmol g^−1^. 2,2,3,3,4,4‐Hexafluoropentane‐1,5‐diol (HFPD, purity ≥98%) was obtained from Tokyo Chemical Industry Co., Ltd. Polylactic acid (PLA) films were supplied by UBS Tape (Hangzhou) Co., Ltd., and copper foil with a thickness of 0.05 mm was purchased from Taizhou Jueyu Metal Materials Co., Ltd. All materials were used as received without further purification.

### 4.2 Preparation of HFPD/TOCNF Composite Films

HFPD/TOCNF composite films were prepared at HFPD‐to‐TOCNF mass ratios of 1:1, 2:1, 3:1, and 4:1 (denoted as HFPD_1_/TOCNF, HFPD_2_/TOCNF, HFPD_3_/TOCNF, and HFPD_4_/TOCNF, respectively). Specifically, 0.11, 0.21, 0.32, and 0.42 g of HFPD were added to 10 mL of a 1.06 wt.% TOCNF aqueous dispersion, respectively. The mixtures were magnetically stirred for 3 h to ensure homogeneity, followed by vacuum degassing to remove entrapped air. The resulting dispersions were cast into polystyrene Petri dishes (35 mm in diameter) and dried at 40 °C for 48 h in a conventional oven without humidity control to obtain composite films. Fabrication of HFPD/TOCNF PENGs: The HFPD/TOCNF composite films were cut into 2.0 cm × 2.0 cm squares. Copper electrodes were attached to both sides of each film, with the electrode area slightly smaller than that of the film to prevent electrical shorting. Conductive wires were connected to the electrodes, and the entire device was encapsulated with a biodegradable PLA film to protect it from environmental electromagnetic interference and mechanical damage, thereby forming the HFPD/TOCNF‐based PENG.

### 4.3 Performance Testing of Piezoelectric Sensors

The electrical output of the PENGs, including the open‐circuit voltage, short‐circuit current, and charging behavior, was measured using a Keithley 6514 electrometer. For data acquisition, the PENGs were connected to a multichannel data acquisition system, and the output signals were recorded and processed using computer‐based software. The assembled piezoelectric sensor was pasted on the throat, wrist, elbow, knuckle, knee, and sole of the foot for sensing tests. Human participant tests (speech, joint motion, Morse code communication, etc.): Written informed consent was obtained from all volunteers. The study was approved by the Academic Committee of Fujian Agriculture and Forestry University, the designated institutional ethics review board.

### 4.4 Characterizations

The morphology of TOCNF was examined by transmission electron microscopy (TEM, Talos F200S G2) operated at 200 kV. Samples were prepared by depositing 10 µL of a 0.01 wt.% TOCNF aqueous suspension onto carbon‐coated copper grids, followed by air‐drying overnight. Crystalline structures were analyzed using X‐ray diffraction (XRD, Bruker D8 Advance) with Cu Kα radiation (λ = 1.54 Å), recorded over a 2θ range of 5–60° at a scanning rate of 5° min^−1^. Surface morphology and elemental distribution were characterized by scanning electron microscopy (SEM, Bruker Nano) equipped with energy‐dispersive X‐ray spectroscopy (EDS) at an accelerating voltage of 5 kV. X‐ray photoelectron spectroscopy (XPS, Thermo Scientific K‐Alpha) was employed to determine elemental composition and chemical states. Attenuated total reflectance Fourier‐transform infrared (ATR‐FTIR) spectra were collected over the wavenumber range of 4000–600 cm^−1^. Differential scanning calorimetry (DSC) measurements were performed using a NETZSCH DSC 214 Polyma under an argon atmosphere. Samples were heated and cooled at a rate of 10 K min^−1^ in aluminum crucibles. Variable temperature infrared spectrometry (VT‐FTIR) measurements were conducted using a Nicolet iS50 spectrometer (Thermo Fisher Scientific). The samples were heated from 20°C to 50°C at a rate of 10°C·min^−1^, and then from 50°C to 90°C at 5°C·min^−1^. During the heating process, VT‑FTIR spectra were collected in the range of 4000–1000 cm^−1^. A total of 11 sets of spectra were recorded during the heating process. Raman spectra were acquired using a Raman imaging microscope (Horiba LabRAM HR Evolution, Japan). Solid‐state ^13^C nuclear magnetic resonance (^13^C NMR) spectra were recorded at room temperature on a Bruker Avance Neo 400WB spectrometer operating at 100 MHz. Mechanical properties were evaluated using a universal testing machine (Instron 3365). Film specimens (5 mm × 30 mm) were stretched at a constant rate of 2 mm min^−1^ until fracture, with three replicates tested for each composition. Bending cycle tests were performed using a universal testing machine (UTM5305H). The film samples underwent bending cycles at a constant speed of 100 mm/min and a bending strain of 30%. The *d*_33_ was measured using a quasi‐static *d*_33_ meter (ZJ‐4AN, Institute of Acoustics, Chinese Academy of Sciences) without poling treatment. Atomic force microscopy (AFM) measurements were performed in piezoresponse force microscopy (PFM) mode using a Bruker Dimension Icon to map surface piezoelectric responses. The output voltage and current of the HFPD/TOCNF PENG were accurately measured using a Keithley 6514 meter system.

### 4.5 DFT Calculation

All calculations were performed using the Gaussian 16 software package. Geometry optimizations and electronic structure calculations were carried out using the B3LYP functional with the def2‐SVP basis set, including empirical dispersion corrections (GD3(BJ)). Frequency calculations were conducted at the same level of theory to confirm that the optimized structures correspond to true minima on the potential energy surface, with no imaginary frequencies observed.

The basis set superposition error (BSSE) was corrected using the standard counterpoise method. The binding energy (*E*_b_) was calculated according to the following equation:

$$E_{b}=E_{A-B}-E_{A}-E_{B}+E_{\mathrm{BSSE}}$$

where *E*_A_, *E*_B_, and *E*_AB_ were the total energies of A, B, and the AB complex, respectively. *E*_BSSE_ is the basis set superposition error correction energy, used for correcting the geometries and interaction energies.

## Author Contributions

**Tingshuai Luo**: Data curation, Investigation, Methodology, Writing – original draft. **Tian Xiao**: Data curation, Investigation. **Luyu Zhang**: Investigation. **Bailang Zhang**: Validation. **Shengchang Lu**: Methodology, Validation, Writing – review & editing. **He Xiao**: Validation. **Jianguo Li**: Validation, Writing – review & editing. **Kai Liu**: Validation. **Chaoji Chen**: Methodology, Supervision, Writing – review & editing. **Hui Wu**: Conceptualization, Supervision, Writing – review & editing.

## Conflicts of Interest

The authors declare no conflicts of interest.

## Supporting information




**Supporting File 1**: advs77780‐sup‐0001‐SuppMat.docx.

**Supporting File 2**: advs77780‐sup‐0002‐VideoS1.mp4.

**Supporting File 3**: advs77780‐sup‐0003‐VideoS2.mp4.

**Supporting File 4**: advs77780‐sup‐0004‐VideoS3.mp4.

**Supporting File 5**: advs77780‐sup‐0005‐VideoS4.mp4.

**Supporting File 6**: advs77780‐sup‐0006‐VideoS5.mp4.

**Supporting File 7**: advs77780‐sup‐0007‐VideoS6.mp4

## Data Availability

The data that support the findings of this study are available on request from the corresponding author. The data are not publicly available due to privacy or ethical restrictions.
