# Supplementary material for: A Recyclable and Biodegradable High‐Performance Piezoelectric Nanogenerator for Self‐Powered Biosensing and Assistive Human‐Machine Interaction
Source: Adv Sci (Weinh). 2026 Sep 27:e77780. Online ahead of print. doi: 10.1002/advs.77780 (PMC13616377; doi:10.1002/advs.77780)
Supplement: Supplementary file 1 [file ADVS-9999-e77780-s004.docx]

# Supplementary Information for

# **A Recyclable and Biodegradable High-Performance Piezoelectric Nanogenerator for Self-Powered Biosensing and Assistive Human–Machine Interaction**

Tingshuai Luo^a^, Tian Xiao^a^, Luyu Zhang^a^, Bailang Zhang^b^, Shengchang Lu^a,*^, He Xiao^a^, Jianguo Li^a,*^, Kai Liu^a^, Chaoji Chen^b,*^, Hui Wu^a,*^

^a^ *College of Material Engineering, Fujian Agriculture and Forestry University, Fuzhou 350108, P. R. China*

^b^ *Hubei Biomass-Resource Chemistry and Environmental Biotechnology Key Laboratory, Hubei Provincial Engineering Research Center of Emerging Functional Coating Materials, School of Resource and Environmental Sciences, Wuhan University, Wuhan 430079, China*

* Corresponding Authors: sciencelu@fafu.edu.cn (S. Lu), [jianguolicn@fafu.edu.cn](mailto:jianguolicn@fafu.edu.cn) (J. Li), [chenchaojili@whu.edu.cn](mailto:chenchaojili@whu.edu.cn) (C. Chen), [wuhui@fafu.edu.cn](mailto:wuhui@fafu.edu.cn) (H. Wu)

# **Supporting text methods**

# **Cytotoxicity assay**

Mouse embryonic fibroblast NIH/3T3 cells were purchased from Qisai Biotechnology (Wuhan, China). Cells were cultured at 37 °C in a humidified atmosphere containing 5% CO_2_ using growth medium supplemented with 10% fetal bovine serum and 1% penicillin–streptomycin. For cell viability evaluation, NIH/3T3 cells were seeded into 96-well plates at a density of 5 × 10^3^ cells per well and cultured for 24 h. The culture medium was then replaced with fresh medium containing HFPD/TOCNF at concentrations of 1, 5, and 10 μg mL⁻¹. After 24 h of incubation, the cells were washed three times with phosphate-buffered saline (PBS), followed by the addition of 10 μL of Cell Counting Kit-8 (CCK-8; Bimake, China) solution to each well. After incubation for 1 h, cell viability was quantified using a microplate reader by measuring the optical density at 450 nm. For live/dead staining, cells were incubated with culture medium containing 10 μg mL⁻¹ HFPD/TOCNF for 24 h and subsequently washed three times with PBS. The cells were then stained with calcein-AM and propidium iodide (PI) (Solarbio, China) according to the manufacturer’s instructions and imaged using a laser scanning confocal microscope (Leica SP8).

**In vivo study**

Animal experiments (subcutaneous implantation in rats and intravenous injection in mice): All procedures were approved by the Institutional Animal Care and Use Committee of Fujian Agriculture and Forestry University (Approval No.: PZCASFAFU26107). The experiments were conducted in accordance with the NIH Guide for the Care and Use of Laboratory Animals.

To evaluate the in vivo biosafety of HFPD and its composites, mice were administered HFPD via tail vein injection at a dose of 80 mg kg^-1^. At 24 h and 48 h post-injection, major organs, including the heart, liver, spleen, lungs, and kidneys, were harvested following standardized procedures. The collected tissues were processed according to conventional histological protocols and stained with hematoxylin and eosin (H&E) for histopathological analysis. For in vivo signal acquisition, the skin over designated regions of the thigh and chest was disinfected using an alcohol-based scrub, followed by the creation of an approximately 2 cm skin incision. The HFPD/TOCNF PENG device (5 mm × 10 mm) was implanted between the epithelial layer and the underlying muscle tissue to record limb motion signals. The electrical output of the implanted device was continuously recorded using a LabVIEW-based data acquisition system. For respiratory motion monitoring, the HFPD/TOCNF PENG device was similarly placed on the pectoralis major muscle of rats to collect respiration-related signals.

# **Supplementary Figures**

**Figure S1.** **SEM images of the HFPD/TOCNF composite films.** (a) TOCNF, (b) HFPD_1_/TOCNF, (c) HFPD_3_/TOCNF, (d) HFPD_4_/TOCNF, and (e) corresponding F-element mapping of the HFPD_2_/TOCNF film.

# **Figure S2.** XPS spectra of the TOCNF and HFPD_2_/TOCNF.

**Figure S3.** (a) DSC results of HFPD/TOCNF composite films during heating. (b) DSC results of HFPD/TOCNF composite films during cooling.

**Figure S4.** (a-b) VT-FTIR spectra of TOCNF. (c-d) VT-FTIR spectra of HFPD_2_/TOCNF.

**Figure S5.** Raman spectra for the TOCNF and HFPD_2_/TOCNF.

**Figure S6.** Work of fracture of TOCNF and HFPD/TOCNF composite films.

**Figure S7.** (a) Amplitude and (b) phase diagram of TOCNF under the applied voltage of 10 V. (c) Amplitude-voltage curve (butterfly loop) and (d) phase-voltage curve (hysteresis loop) of TOCNF. (e) Amplitude and (f) phase diagram of HFPD/TOCNF under the applied voltage of 10 V.

**Figure S8.** Working mechanism of HFPD/TOCNF PENG device.

**Figure S9.** **The output power and power density of HFPD/TOCNF PENGs.** (a) The output power and power density of HFPD/TOCNF PENGs. (b) *V*_OC_ and (c) *I*_SC_ of TOCNF and HFPD_2_/TOCNF PENGs with different loading stress.

**Figure S10.** Capacitor charging profiles under a vertical compressive stress of 100 kPa.

# **Supplementary Table:**

**Table S1.** Performance comparison for piezoelectric HFPD/TOCNF and PVDF film[1-4]

|  | HFPD/TOCNF | PVDF |
| --- | --- | --- |
| Non-toxic  processing | Water  +++++ | N, N-dimethylformamide (DMF), acetone (AR)  + |
| Degradability | 20 days  +++++ | Almost non-degradable  + |
| Recyclability | Stirring and re-casting  +++++ | Mechanical  ++ |
| Flexibility | 0.64~1.03 Gpa  ++++ | 0.23~1.61 GPa  +++++ |
| Biocompatibility | Cell viability: 101%  +++++ | Cell viability: 98%  +++++ |

| Materials | *d*_33_  (pC·N^−1^) | *V*_OC_  (V) | Biodegradability? | Recyclability? | Ref. |
| --- | --- | --- | --- | --- | --- |
| TOCNF | 2.55 | ~1 | yes | yes | Our work |
| TOCNF/MoS₂ | 31 | ~6 | no | no | [5] |
| CNF@ZnO | ~10 | ~5 | no | no | [2] |
| CNF/PVDF/BaTiO_3_ | ~5 | ~4 | no | no | [6] |
| CNF/BNNTs | ~11 | — | no | no | [7] |
| CNF-chitosan | 4 | — | yes | no | [8] |
| PVDF/CNC | ~26 | 12 | no | no | [9] |
| HFPD/TOCNF | ~14 | ~8 | yes | yes | Our work |

**Table S2.** Comparison of piezoelectric HFPD/TOCNF with existing cellulose-based piezoelectric materials

**Table S3.** Performance comparison of the HFPD/TOCNF film with reported piezoelectric materials in terms of d33 and output voltage.

| Materials | *d*_33_  (pC·N^−1^) | *V*_OC_  (V) | Ref. |
| --- | --- | --- | --- |
| PVDF/TPPC | 2.15 | 6.62 | [10] |
| PVDF/4Ag-Pbt | 8.2 | 10 | [11] |
| PVDF/BiCl3/ZnO | 3.8 | 12 | [12] |
| CNF@ZnO | 10.7 | 4.52 | [2] |
| Oxidized fiber/ZnO | 3.8 | 5.5 | [13] |
| F-SS/ZnO/PVA | 12 | 0.85 | [14] |
| CNF/PVDF/CNF-BaTiO_3_ | 5.25 | 4.4 | [6] |
| BTO@PCL | 3 | 5.58 | [15] |
| γ-glycine/PVA | 10.4 | 0.22 | [16] |
| CNC/PEG | 5.33 | 1.76 | [17] |
| Fish skin | 3 | 0.2 | [18] |
| PLLA | 1.75 | 1 | [19] |
| glycine-PVA film | 5.3 | 4.1 V | [20] |
| β-glycine/polyvinylpyrrolidone films | 9.6 | ~3.6 V | [21] |
| HFPD/TOCNF | ~14 | ~8 | Our work |

# **Supplementary Video:**

# **Video S1.** The piezoelectric signal for the Morse code “SOS”.

# **Video S2.** The piezoelectric signal for the Morse code “COLD”.

# **Video S3.** The piezoelectric signal for the Morse code “PAIN”.

# **Video S4.** The piezoelectric signal for the Morse code “HELP”.

# **Video S5.** The piezoelectric signal for the Morse code “COLD”.

# **Video S5.** Piezoelectric signals implanted in the quadriceps muscle of mice.

# **Video S6.** Voltage signals implanted into the pectoralis major muscle of mice.

**Supporting references**

[1] J. Xiong, L. Wang, F. Liang, et al., "Flexible Piezoelectric Sensor Based on Two-Dimensional Topological Network of PVDF/DA Composite Nanofiber Membrane," *Advanced Fiber Materials* 6(4) (2024): 1212-1228, <https://doi.org/10.1007/s42765-024-00415-7>.

[2] Q. Zhu, X. Song, X. Chen, et al., "A high performance nanocellulose-PVDF based piezoelectric nanogenerator based on the highly active CNF@ZnO via electrospinning technology," *Nano Energy* 127 (2024): 109741, <https://doi.org/https://doi.org/10.1016/j.nanoen.2024.109741>.

[3] S. Panda, S. Hajra, H. Jeong, et al., "Biocompatible CaTiO_3_-PVDF composite-based piezoelectric nanogenerator for exercise evaluation and energy harvesting," *Nano Energy* 102 (2022): 107682, <https://doi.org/https://doi.org/10.1016/j.nanoen.2022.107682>.

[4] X. Du, Z. Zhou, Z. Zhang, et al., "Porous, multi-layered piezoelectric composites based on highly oriented PZT/PVDF electrospinning fibers for high-performance piezoelectric nanogenerators," *Journal of Advanced Ceramics* 11(2) (2022): 331-344, <https://doi.org/10.1007/s40145-021-0537-3>.

[5] T. Wu, Y. Song, Z. Shi, et al., "High-performance nanogenerators based on flexible cellulose nanofibril/MoS2 nanosheet composite piezoelectric films for energy harvesting," *Nano Energy* 80 (2021): 105541, <https://doi.org/https://doi.org/10.1016/j.nanoen.2020.105541>.

[6] M. Li, B. Jiang, S. Cao, et al., "Flexible cellulose-based piezoelectric composite membrane involving PVDF and BaTiO_3_ synthesized with the assistance of TEMPO-oxidized cellulose nanofibrils," *RSC Advances* 13(15) (2023): 10204-10214, <https://doi.org/10.1039/D3RA00604B>.

[7] M.S. Tahir, J.W. Park, J.-G. Han, et al., "Flexible Piezoelectric Papers Fabricated Using Amine-Modified Cellulose Nanofibers and Boron Nitride Nanotubes Nanocomposites," *ACS Applied Polymer Materials* 7(7) (2025): 4394-4403, <https://doi.org/10.1021/acsapm.5c00111>.

[8] A. Hänninen, E. Sarlin, I. Lyyra, et al., "Nanocellulose and chitosan based films as low cost, green piezoelectric materials," *Carbohydrate Polymers* 202 (2018): 418-424, <https://doi.org/https://doi.org/10.1016/j.carbpol.2018.09.001>.

[9] L. Pan, Y. Wang, Q. Jin, et al., "Self-poled PVDF/recycled cellulose composite fibers utilizing cellulose nanocrystals to induce PVDF β-phase formation through wet-spinning as a flexible fabric piezoelectric sensor," *Chemical Engineering Journal* 479 (2024): 147742, <https://doi.org/https://doi.org/10.1016/j.cej.2023.147742>.

[10] H. Pei, Y. Xie, Y. Xiong, et al., "A novel polarization-free 3D printing strategy for fabrication of poly (Vinylidene fluoride) based nanocomposite piezoelectric energy harvester," *Composites Part B: Engineering* 225 (2021): 109312, <https://doi.org/https://doi.org/10.1016/j.compositesb.2021.109312>.

[11] C. Shuai, G. Liu, Y. Yang, et al., "A strawberry-like Ag-decorated barium titanate enhances piezoelectric and antibacterial activities of polymer scaffold," *Nano Energy* 74 (2020): 104825, <https://doi.org/https://doi.org/10.1016/j.nanoen.2020.104825>.

[12] D. Zhang, X. Zhang, X. Li, et al., "Enhanced piezoelectric performance of PVDF/BiCl_3_/ZnO nanofiber-based piezoelectric nanogenerator," *European Polymer Journal* (166) (2021): 110956, <https://doi.org/10.1016/j.eurpolymj.2021.110956>.

[13] X. Chen, Q. Zhu, D. Li, et al., "A wood fiber-based multifunctional generator for multiple environmental energy harvesting," *Chemical Engineering Journal* 500 (2024): 156026, <https://doi.org/https://doi.org/10.1016/j.cej.2024.156026>.

[14] Q. Lv, S. Chen, D. Luo, et al., "An Implantable and Degradable Silk Sericin Protein Film Energy Harvester for Next-Generation Cardiovascular Electronic Devices," *Advanced Materials* 37(22) (2025): 2413610, <https://doi.org/https://doi.org/10.1002/adma.202413610>.

[15] Y. Liu, Z. Zhang, Z. Zhao, et al., "An Easy Nanopatch Promotes Peripheral Nerve Repair through Wireless Ultrasound-Electrical Stimulation in a Band-Aid-Like Way," *Advanced Functional Materials* 34(44) (2024): 2407411, <https://doi.org/https://doi.org/10.1002/adfm.202407411>.

[16] H. Xue, J. Jin, Z. Tan, et al., "Flexible, Biodegradable Ultrasonic Wireless Electrotherapy Device Based on Highly Self-Aligned Piezoelectric Biofilms," *Science Advances* 10(22) (2024): eadn0260, <https://doi.org/10.1126/sciadv.adn0260>.

[17] S. Li, C. Liu, W. Chen, et al., "Negative Poisson's Ratio in Cellulose Nanocrystal Aerogels for Enhanced Sports Piezoelectric Sensing," *Advanced Functional Materials* 35(29) (2025): 2418425, <https://doi.org/https://doi.org/10.1002/adfm.202418425>.

[18] S.K. Ghosh, D. Mandal, "Sustainable Energy Generation from Piezoelectric Biomaterial for Noninvasive Physiological Signal Monitoring," *ACS Sustainable Chemistry & Engineering* 5(10) (2017): 8836-8843, <https://doi.org/10.1021/acssuschemeng.7b01617>.

[19] E.J. Curry, K. Ke, M.T. Chorsi, et al., "Biodegradable Piezoelectric Force Sensor," *Proceedings of the National Academy of Sciences* 115(5) (2018): 909-914, <https://doi.org/10.1073/pnas.1710874115>.

[20] F. Yang, J. Li, Y. Long, et al., "Wafer-scale heterostructured piezoelectric bio-organic thin films," *Science (1979)* 373(6552) (2021): 337-342, <https://doi.org/doi:10.1126/science.abf2155>.

[21] X. Li, Z. Zhang, Y. Zheng, et al., "One-Step High-Speed Thermal-Electric Aerosol Printing of Piezoelectric Bio-Organic Films for Wirelessly Powering Bioelectronics," *Science Advances* 10(43) (2024): eadq3195, <https://doi.org/> 10.1126/sciadv.adq3195.
